# The relationship between positive coaching behaviors and sport engagement among young elite wrestlers: the mediating role of sport enjoyment

**DOI:** 10.3389/fpsyg.2026.1860935

**Published:** 2026-08-03

**Authors:** Burhan Özkurt, Yasin Altın, Hüseyin Fatih Küçükibiş, Oğuzhan Çalı, Rahmi Yıldız

**Affiliations:** Faculty of Sports Sciences, Sivas Cumhuriyet University, Sivas, Türkiye

**Keywords:** coach–athlete relationship, mediation, positive coaching behaviors, sport engagement, sport enjoyment, young elite wrestlers

## Abstract

This study examined the relationship between positive coaching behaviors and sport engagement and the mediating role of sport enjoyment in this relationship. The study employed a quantitative cross-sectional design, and the data were analyzed using structural equation modeling (SEM). The sample consisted of 438 elite male wrestlers aged 18–21 years who were actively competing in Türkiye (Greco-Roman, *n* = 247; freestyle, *n* = 191). Data were collected through a survey, and all statistical analyses were performed using R 4.3.2. Positive coaching behaviors were significantly associated with sport engagement (*β* = 0.239) and sport enjoyment (*β* = 0.583), while sport enjoyment was significantly associated with sport engagement (*β* = 0.663; all *p* < 0.001). The model also indicated a significant indirect effect of positive coaching behaviors on sport engagement through sport enjoyment (*β* = 0.387, *p* < 0.001). These findings suggest that sport engagement among young elite wrestlers is associated not only with perceived positive coaching behaviors but also with the enjoyment derived from the sport experience. Accordingly, sport enjoyment should be considered an important psychological factor when examining athletes’ sport engagement.

## 1 Introduction

Sport experience is often evaluated through visible outcomes, while the processes that produce these outcomes are largely overlooked (Akkaya, 2019). However, athletes’ performance is not the only important consideration; how they achieve it, how they experience the process, and the extent to which they are willing to continue participating in sport are at least as important as the outcome itself. In this respect, the sport environment is not only a field in which physical performance is displayed, but also an interactional space in which meanings, expectations, and emotional responses are shaped (Campo et al., 2019; Tamminen and Bennett, 2017). Within this interactional space, coaches are among the most powerful social actors who directly influence athletes’ cognitive, emotional, and behavioral processes (Jowett and Poczwardowski, 2007; Jowett, 2025).

Coaches are not only technical experts who support athletes’ performance development, but also key determinants that shape the emotional and motivational quality of the sport experience (Mageau and Vallerand, 2003; van Kleef et al., 2019). Coach–athlete interaction directly influences how athletes perceive their experience and how they respond emotionally to this process through communication style, the quality of feedback, and the behaviors displayed (Côté and Gilbert, 2009; Cassidy et al., 2023). In particular, supportive and development-oriented coaching behaviors contribute to making the sport experience more positive by helping athletes feel more competent and valued (Gencer and Öztürk, 2018; Lafrenière et al., 2011; Ruiz et al., 2021), whereas controlling and pressuring approaches may affect this process negatively (Isoard-Gautheur et al., 2016; Liu et al., 2025). This makes it even more important to understand the role of coaching behaviors in athletes’ enjoyment of sport and their levels of sport engagement.

### 1.1 Theoretical background and hypothesis development

Self-Determination Theory (SDT) and the Sport Commitment Model provide important theoretical frameworks for explaining these relationships. According to SDT, individuals’ motivational and emotional processes are closely related to the extent to which their basic psychological needs, such as autonomy, competence, and relatedness, are satisfied (Deci and Ryan, 2000). In the sport context, coaches play a critical role in supporting or thwarting these needs (Hollembeak and Amorose, 2005; Li et al., 2025; Ruiz et al., 2021). The Sport Commitment Model, in turn, explains athletes’ psychological commitment to sport and identifies sport enjoyment as one of the strongest psychological determinants of this commitment (Scanlan et al., 1993). In line with these theoretical frameworks, the relationships examined in the present study and the corresponding hypotheses are presented below in sequence.

### 1.2 Positive coaching behaviors and sport engagement

Commitment is conceptualized as the psychological bond that an individual develops toward a particular person, group, institution, or activity; this bond reflects dimensions such as belonging, identification, and the desire to maintain the relationship (Meyer and Allen, 1991). Sport commitment, in turn, refers to the psychological attachment that an individual develops toward a particular sport, team, or sporting activity (Scanlan et al., 1993). Therefore, sport commitment cannot be considered independently of the social interactions established within the sport environment. Indeed, the social environment, particularly coaching behaviors, may influence how athletes evaluate the sport environment and the extent to which they become attached to it (Gu et al., 2023; Kassim and Boardley, 2018). In particular, positive coaching behaviors are thought to strengthen sport commitment by making the sport experience more supportive and meaningful (Altın et al., 2023; Ji et al., 2026). Based on these theoretical and empirical explanations, the following hypothesis was formulated:

*H1*: There is a positive and significant relationship between positive coaching behaviors and sport engagement.

### 1.3 Positive coaching behaviors and sport enjoyment

Enjoyment is defined as the pleasure, satisfaction, and positive emotional experiences that individuals derive from an activity, experience, or situation (Kimiecik and Harris, 1996; Özkurt et al., 2022a; Scanlan et al., 1993). Sport enjoyment, in turn, refers to the positive emotions individuals experience while engaging in sport or participating in sporting activities, as well as the psychological satisfaction they derive from this process (Özkurt et al., 2022a,b; Scanlan et al., 1993). In this respect, sport enjoyment is not merely a momentary feeling of pleasure but also an important indicator of the extent to which individuals evaluate their sport experience as positive and meaningful (McCarthy et al., 2008).

Enjoyment experienced in the sport environment may be influenced not only by individual characteristics but also by the quality of the social environment. In this context, coaching behaviors may be associated with the enjoyment athletes derive from sport through their connection with how athletes perceive the sport environment and their sport experiences (Bugten et al., 2025; Gardner et al., 2016). Coaches’ supportive, constructive, and development-oriented behaviors may contribute to athletes evaluating their sport experiences more positively and deriving greater enjoyment from sport (Kim et al., 2021; Ruiz et al., 2019). Based on these theoretical and empirical explanations, the following hypothesis was developed:

*H2*: There is a positive and significant relationship between positive coaching behaviors and sport enjoyment.

### 1.4 Sport enjoyment and sport engagement

Sport enjoyment refers to positive emotional responses to the sport experience, such as pleasure, liking, fun, and satisfaction (Özkurt et al., 2022a,b; Scanlan et al., 1993). This experience may arise situationally during a particular training session or competition (Çınar-Medeni et al., 2025), or it may develop into a more general and enduring positive evaluation of sport through repeated sporting experiences (Özkurt et al., 2022a,b). Perceiving the sport experience as enjoyable and personally valuable may make the activity more attractive and worth continuing for the athlete, thereby strengthening the psychological bond with sport (Özkurt, 2023).

In the Sport Commitment Model, sport enjoyment is considered one of the fundamental psychological resources that support the desire and resolve to continue sport participation (Scanlan et al., 1993; Berki et al., 2026). Accordingly, individuals who derive greater enjoyment from their sport experiences are expected to develop a stronger psychological bond with sport and a greater desire to continue their participation. Empirical studies have also shown that sport enjoyment is positively associated with motivation, sport commitment, and the tendency to continue sport participation (Braga-Pereira et al., 2024; Garcia-Mas et al., 2010; Weiss et al., 2001). Based on these theoretical and empirical foundations, the following hypothesis was developed:

*H3*: There is a positive and significant relationship between enjoyment in sport and sport engagement.

### 1.5 The mediating role of sport enjoyment

A coach is not only responsible for guiding an athlete’s technical, tactical, and physical development but is also one of the key actors shaping the social, emotional, and motivational quality of the sport environment (Jowett, 2005; Jowett and Poczwardowski, 2007; Short and Short, 2005). A coach who listens to athletes, values their development, treats them fairly, and provides constructive feedback in response to mistakes may contribute to creating a safer and more supportive sport environment (Altın et al., 2023). According to Self-Determination Theory, such social conditions facilitate more positive motivational and emotional experiences by supporting athletes’ needs for autonomy, competence, and relatedness (Deci and Ryan, 2000; Mageau and Vallerand, 2003). Indeed, coach-provided autonomy support has been shown to be associated with sport enjoyment through psychological need satisfaction and self-determined motivation (Álvarez et al., 2009).

In a supportive environment, athletes may perceive training sessions and competitions not merely as tasks to be completed but as experiences in which they can develop, feel a sense of belonging, and enjoy participating. This positive evaluation may enhance sport enjoyment. Sport enjoyment, in turn, may strengthen athletes’ desire to continue participating in sport and their psychological bond with sport by making the sport experience more attractive and personally valuable. The Sport Commitment Model, which considers enjoyment one of the fundamental psychological resources supporting continued sport participation, is also consistent with this explanation (Scanlan et al., 1993). Findings showing that coach leadership is associated with both sport enjoyment and sport commitment provide empirical support for this proposed indirect process (Bandura and Kavussanu, 2018; Braga-Pereira et al., 2024).

From this perspective, rather than being someone who directly binds athletes to sport, the coach may be viewed as a social actor who contributes to creating a supportive environment in which sport engagement can develop. Sport enjoyment may therefore be considered one of the mediating processes that explain how this environment is transformed into a stronger psychological bond with sport. Based on these theoretical and empirical foundations, the following hypothesis was developed:

*H4*: Sport enjoyment mediates the relationship between positive coaching behaviors and sport engagement.

In the literature, the relationships between coaching behaviors, enjoyment in sport, and sport engagement have been examined across different athlete samples. However, in studies where these variables are investigated within the same model, it is observed that empirical research directly testing the mediating role of enjoyment in sport in the relationship between positive and supportive coaching behaviors and sport engagement is limited (Álvarez et al., 2009; Murillo et al., 2022; O’Neil and Hodge, 2020). A significant portion of the existing studies has addressed the coach effect through motivational outcomes such as basic psychological need satisfaction and motivational regulations, whereas studies incorporating enjoyment in sport into the model are relatively scarce (Granero-Gallegos et al., 2017; Mossman et al., 2022). This situation points to a research gap in the literature in terms of both empirical investigation and sample representation (Miles, 2017). Therefore, it is important to examine the relationships among positive coaching behaviors, sport enjoyment, and sport engagement.

Examining these relationships among young elite wrestlers is particularly important. Athletes in developmental stages may be especially responsive to coaching influence because they are more sensitive to feedback from their social environment and their sport-related emotions, attitudes, and engagement are still developing (Keegan et al., 2010; Stein et al., 2012). Among elite athletes, high performance expectations, intensive training loads, and persistent pressure to succeed may further increase the importance of psychological processes such as sport enjoyment and sport engagement (Scott et al., 2025). In addition, wrestling provides a particularly relevant context for examining the effects of coaching behaviors because of its high physical and psychological demands, disciplined training structure, and close coach–athlete interactions (Terekli et al., 2026). Accordingly, the present study examined the relationships among positive coaching behaviors, sport enjoyment, and sport engagement in young elite wrestlers, as well as the mediating role of sport enjoyment in the relationship between positive coaching behaviors and sport engagement.

## 2 Materials and methods

### 2.1 Research method

This study was conducted within the framework of a quantitative research approach based on a cross-sectional design, and the data were analyzed using Structural Equation Modeling (SEM). In the study, the relationship between positive coaching behaviors and athletes’ levels of sport engagement was examined; additionally, the mediating role of enjoyment in sport in this relationship was evaluated. Structural equation modeling is a powerful statistical method for analyzing direct and indirect relationships among variables and for testing mediation effects (Kline, 2015). This model enables a comprehensive examination of the conceptual framework of the study by simultaneously analyzing the relationships between observed and latent variables. The independent, dependent, and mediating variables of the study are as follows:

- Independent variable: Positive coaching behaviors
- Dependent variable: Sport engagement
- Mediating variable: enjoyment

**Figure 1:**
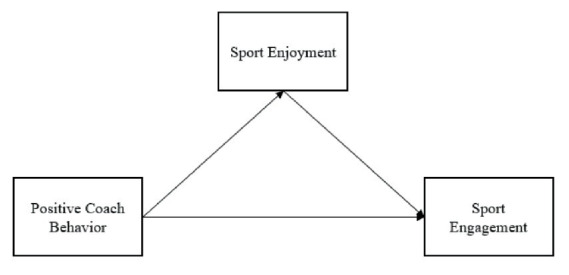
Model including research hypotheses.

Within the framework of this model, the direct relationship between the positive behaviors displayed by coaches and athletes’ levels of sport engagement was examined, and the mediating role of enjoyment in sport in this relationship was tested. A schematic representation of the research model is presented in Figure 1.

### 2.2 Participants

The study sample consisted of elite male athletes aged between 18 and 21 who competed in Greco-Roman and Freestyle wrestling. The participants were selected from licensed athletes who actively competed in official tournaments and competitions organized within the activity program of the Turkish Wrestling Federation.

In determining the sample size, recommended sample adequacy criteria for Structural Equation Modeling (SEM) analyses were taken into consideration. Kline (2015) states that, in SEM analyses based on maximum likelihood estimation, sample size should be evaluated in relation to the number of parameters included in the model. In this context, the literature suggests that approximately 10 participants per parameter is the minimum, whereas 20 participants per parameter is considered a more ideal ratio. Additionally, sample sizes of approximately 200 or more are generally regarded as sufficient in SEM studies; however, larger samples may be required as model complexity increases (Kline, 2015).

Accordingly, a total of 438 male athletes were included in the study (Greco-Roman *n* = 247; Freestyle *n* = 191). A purposive sampling method was employed, and athletes who met the criteria determined in terms of the research variables were included in the study. In this regard, the inclusion criteria were that participants were actively engaged in wrestling, were licensed athletes, competed at an elite level, were between the ages of 18 and 21, and voluntarily agreed to participate in the study.

### 2.3 Data collection instruments

In this study, the relationship between positive coaching behaviors and athletes’ levels of sport engagement was examined, and the mediating role of enjoyment in this process was evaluated. Accordingly, the Personal Information Form, the Coaching Behavior Assessment Scale for Athletes (CBAS-A), the Sport Engagement Scale, and the Physical Activity Enjoyment Scale were used as data collection instruments.

#### 2.3.1 Personal information form

A Personal Information Form developed by the researchers was used to determine the demographic characteristics of the participants. The form included items related to age, educational level, years of sport participation, wrestling style (Greco-Roman/Freestyle), and level of achievement in their sport careers. Of the participants, 62.8% had a high school level of education, while 37.2% had a university-level education. The participants’ sport experience ranged between 6 and 8 years. Additionally, the participants had various achievements at national and international levels.

#### 2.3.2 Coaching Behavior Scale for Sport (CBS-S)

The Coaching Behavior Scale for Sport (CBS-S), originally developed by Côté et al. (1999) and adapted into Turkish by Yapar and İnce (2016), was used to evaluate coaching behaviors based on athletes’ perceptions. The original form of the scale consists of 47 items and 6 sub-dimensions. In the present study, only the sub-dimension assessing positive coaching behaviors was utilized. This sub-dimension consists of 7 items. The scale was rated on a 7-point Likert scale ranging from 1 (never) to 7 (always).

To assess the construct validity of the subscale in the present sample, confirmatory factor analysis was conducted. The one-factor model demonstrated an acceptable fit to the data: CFI = 0.99, TLI = 0.98, IFI = 0.99, RMSEA = 0.08, 90% CI [0.05, 0.10], and SRMR = 0.02. Standardized factor loadings ranged from 0.62 to 0.89 and were statistically significant (*p* < 0.001). These findings provided support for the one-factor structure and construct validity of the Positive Coaching Behaviors subscale in the present sample.

#### 2.3.3 Sport engagement scale

To determine athletes’ levels of sport engagement, the Sport Engagement Scale, originally developed by Schaufeli and Bakker (2003), revised by Guillén and Martínez-Alvarado (2014), and adapted into Turkish by Kayhan et al. (2020), was used. The scale is a 10-item measurement instrument consisting of two sub-dimensions, namely Focus and Vigor. It was rated on a 7-point Likert scale ranging from 1 (never) to 7 (always), and it was stated that the scale does not include any reverse-coded items.

To assess the construct validity of the scale in the present sample, confirmatory factor analysis was conducted. The two-factor model demonstrated an acceptable fit to the data: CFI = 0.95, TLI = 0.94, IFI = 0.95, RMSEA = 0.08, 90% CI [0.07, 0.09], SRMR = 0.04, and RMR = 0.04. The standardized factor loadings ranged from 0.54 to 0.82 and were statistically significant (*p* < 0.001). These findings supported the two-factor structure and construct validity of the Sport Engagement Scale in the present sample.

#### 2.3.4 Enjoyment scale

To assess the extent to which athletes enjoy physical activity, the Physical Activity Enjoyment Scale, originally developed by Mullen et al. (2011) and adapted into Turkish by Özkurt et al. (2022b), was used. The scale consists of 8 unidimensional items evaluating perceived enjoyment and feelings of pleasure. It was rated on a 7-point Likert-type scale ranging from 1 (strongly disagree) to 7 (strongly agree).

To examine the construct validity of the scale in the present sample, a one-factor confirmatory factor analysis was conducted. The incremental fit indices indicated good model fit (robust CFI = 0.97, robust TLI = 0.96, robust IFI = 0.98), although the robust RMSEA value was relatively high, RMSEA = 0.10, 90% CI [0.07, 0.11]. Standardized factor loadings ranged from 0.82 to 0.90 and were statistically significant (*p* < 0.001), generally supporting the unidimensional structure of the scale.

#### 2.3.5 Validity and reliability analysis of the scales

The validity and reliability properties of all scales used in this study were re-evaluated on the current sample. The construct validity of the scales was tested using Confirmatory Factor Analysis (CFA), while reliability was assessed through Cronbach’s Alpha and McDonald’s Omega coefficients. In addition, composite reliability (CR) and average variance extracted (AVE) were considered for convergent validity, and relevant criteria were taken into account for discriminant validity. The findings obtained indicated that the measurement instruments used demonstrated acceptable psychometric properties in the sample of elite-level athletes.

### 2.4 Data collection and research ethics

In this study, data were collected between 2024 and 2025 from participants selected among elite-level youth and junior wrestlers in Türkiye. During the data collection process, questionnaire forms were prepared online (via Google Forms) and distributed to the athletes through WhatsApp. The surveys were delivered to the participants through the Turkish Wrestling Federation and relevant club coaches, and detailed information regarding the purpose, methodology, and confidentiality principles of the study was provided. Participation was entirely voluntary, and athletes had the right to withdraw from the study at any time. The data collection process was completed within approximately 4 to 6 weeks.

The study was conducted in accordance with scientific ethical principles and relevant regulations. The Social Sciences Scientific Research Ethics Committee of Sivas Cumhuriyet University approved the study as ethically appropriate with its decision dated 07.03.2025 and numbered 2 (Document Date and Number: 11.03.2025–539,878), and data collection commenced after obtaining the necessary permissions. Informed consent was obtained from all participants through an informed consent form, and their personal data were protected and anonymized. The identities of the athletes were kept confidential, were not shared with any third parties under any circumstances, and the collected data were analyzed solely for scientific purposes. This study was conducted in accordance with the Declaration of Helsinki and the Personal Data Protection Law (KVKK, Law No. 6698), with utmost care given to protecting participants’ rights, and all procedures were carried out in full compliance with ethical standards.

### 2.5 Data analysis

The data obtained within the scope of the study were evaluated using descriptive statistics. The hypotheses were tested using a structural regression model. Before testing the hypotheses, assumption checks related to the model were conducted. Common method bias was assessed using Harman’s single-factor test by establishing single-factor exploratory and confirmatory factor analysis models. When all observed variables in the dataset were included in the analyses under a single factor, the explained variance was found to be 37% (<50%). In addition, in the single-factor confirmatory factor analysis model, the fit indices were found to be low (CFI = 0.54, TLI = 0.51, RMSEA = 0.16 [0.15, 0.16], and SRMR = 0.15). These findings indicate the absence of common method bias.

In the dataset, 16 observations (3.41%) were identified as univariate outliers (standard *z* score > 3.29) and 5 observations (1.05%) as multivariate outliers (Mahalanobis *D*^2^ > *χ*^2^), and were excluded from the analysis. The skewness coefficients of the latent variables ranged between −2.09 and 0.56, and the kurtosis coefficients ranged between −0.99 and 4.73; values violating the univariate normality assumption based on the reference range (±1.5) were observed (Tabachnick and Fidell, 2013). According to Mardia’s test, multivariate normality was not satisfied (*p* < 0.001).

The multivariate correlations among the variables were found to be significant (*χ*^2^ = 4597.928, df = 496, *p* < 0.001). According to the scatterplots, the relationships among variables were linear. Multiple linear regression models were established between latent and observed variables, and VIF and tolerance values were calculated (VIF < 10 and Tol > 0.10). In addition, the squared multiple correlations (SMC) values ranged between 0.28 and 0.87 (<0.90). Accordingly, no multicollinearity or singularity problem was observed.

Since there was a high proportion of multivariate outliers in the dataset and the normality assumption was not met, the structural equation model was estimated using a robust estimation method. To evaluate the validity of the model, convergent and discriminant validity analyses were conducted and interpreted based on reference criteria (see Fornell and Larcker, 1981; Hair et al., 2010; Karagöz, 2019). To assess convergent and discriminant validity, average variance extracted (AVE), composite reliability (CR), √AVE, maximum shared variance (MSV), and average shared variance (ASV) values were calculated and interpreted according to reference criteria (see Fornell and Larcker, 1981; Hair et al., 2010; Karagöz, 2019). In the evaluation of convergent validity, CR > AVE and AVE > 0.50 were taken as the basis. Discriminant validity was interpreted according to the criteria AVE > MSV, AVE > ASV, and √AVE > *r*. The data were analyzed using the R (4.3.2) software and its associated packages. The findings were interpreted with a 95% confidence interval and a 5% margin of error (*α* = 0.05). To reduce the probability of Type I error, Bonferroni correction was applied. The α value was interpreted by dividing it by the number of parameters whose effects on the dependent variable were examined (α / k).

## 3 Results

To examine the prediction of sport engagement, a structural regression model including the direct effect of positive coaching behaviors and the mediating role of enjoyment was tested using the robust maximum likelihood estimation method. The obtained fit indices indicated that the model demonstrated a good fit to the data. Specifically, the values of CFI.robust (0.975), TLI. robust (0.971), NNFI.robust (0.971), and RNI.robust (0.975) indicated a good model fit. In addition, the robust RMSEA value of 0.043 (95% CI [0.03, 0.05]) further supported the adequacy of the model fit. These findings suggest that the proposed structural model demonstrated a good fit to the data (Brown, 2006; Hu and Bentler, 1999). The descriptive statistics, reliability coefficients, and correlations among the variables are presented in Table 1, while the results related to the measurement model are presented in Table 2.

**Table 1:** Descriptive statistics, reliability coefficients, and correlations among the variables.

Variable	Mean	SD	Enjoyment	Engagement	Positive
1. Enjoyment	50.21	8.03	(0.95, 0.97)		
2. Engagement	63.19	7.86	0.76	(0.90, 0.92)	
3. Positive	38.41	5.63	0.58	0.59	(0.92, 0.94)

Reliability coefficients are reported on the diagonal in parentheses as (*α*, *ω*). Positive, Positive Coaching Behaviors; Enjoyment, Sport Enjoyment; Engagement, Sport Engagement.

**Table 2:** Measurement model.

Indicator	Estimate	Std. error	*z*	*p*(>|*z*|)	*β*
Sport enjoyment=~					
e1	1.00				0.84
e2	1.03	0.04	24.38	<0.001	0.88
e3	1.05	0.05	20.42	<0.001	0.87
e4	1.14	0.09	12.38	<0.001	0.75
e5	1.07	0.07	16.38	<0.001	0.83
e6	1.00	0.06	16.28	<0.001	0.89
e7	1.08	0.07	15.34	<0.001	0.80
e8	1.03	0.07	15.66	<0.001	0.84
Sport engagement=~					
en1	1.00				0.60
en2	0.95	0.08	12.24	<0.001	0.66
en3	1.29	0.10	13.62	<0.001	0.81
en4	0.72	0.09	8.40	<0.001	0.62
en5	1.57	0.13	12.23	<0.001	0.71
en6	1.28	0.10	13.39	<0.001	0.80
en7	1.37	0.11	12.19	<0.001	0.79
en8	1.35	0.14	9.99	<0.001	0.77
en9	1.11	0.09	12.24	<0.001	0.75
en10	1.07	0.10	10.48	<0.001	0.55
Positive behavior=~					
p1	1.00				0.86
p2	1.05	0.08	13.72	<0.001	0.79
p3	1.01	0.08	13.50	<0.001	0.84
p4	1.17	0.07	16.66	<0.001	0.88
p5	1.08	0.09	12.65	<0.001	0.82
p6	0.88	0.08	10.68	<0.001	0.64

These findings suggest that the proposed structural model was confirmed and demonstrated a good fit to the data (Brown, 2006; Hu and Bentler, 1999). The results related to the measurement and structural model are presented in Table 2. Overall, these values indicate that the measurement model was validated and that the model showed a satisfactory level of fit with the data (Brown, 2006; Hu and Bentler, 1999). The findings related to the measurement model are presented in Table 2.

According to Table 2, all paths between the latent and observed variables were statistically significant (*p* < 0.001), with standardized path coefficients ranging from 0.55 to 0.89 (0.50 < *β*). These findings indicate that the model demonstrated a good level of construct validity and that the items strongly represented the latent variables of enjoyment, sport engagement, and positive coaching behavior. The findings regarding convergent and discriminant validity are presented in Table 3.

According to Table 3, convergent validity was established for all variables included in the measurement model (AVE > 0.50, CR > AVE). In terms of discriminant validity, the findings generally supported the required criteria (AVE > MSV, AVE > ASV, and √AVE > *r*). The path coefficients representing the relationships among the latent variables are presented in Table 4.

**Table 3:** Convergent and discriminant validity of the measurement model.

Variable	AVE	CR	√AVE	MSV	ASV
1. Enjoyment	0.69	0.94	0.83	0.64	0.33
2. Engagement	0.59	0.90	0.77	0.64	0.35
3. Positive	0.64	0.90	0.80	0.39	0.24

Positive, Positive Coach Behavior, Enjoyment, Sport Enjoyment, Engagement, Sport Engagement.

According to Table 4, a positive and highly significant effect of positive coaching behaviors on enjoyment in sport was found (*β_a_* = 0.583 [0.48, 0.68]; *z* = 7.64, *p* < 0.001). A one-unit increase in positive coaching behaviors was associated with an increase of B(a) = 0.625 points (SE = 0.082) in enjoyment in sport. A positive and highly significant effect of enjoyment in sport on sport engagement was found (*βᵦ* = 0.663 [0.51, 0.82]; *z* = 5.31, p < 0.001). A one-unit increase in enjoyment in sport was associated with an increase of B(b) = 0.458 points (SE = 0.086) in sport engagement.

**Table 4:** Path coefficients for predicting sport enjoyment and sport engagement.

Relationships	Path coefficient	*B*	SE	*z*	*p*(>|*z*|)	*Β*	95% CI	
							Low	High
Positive → Enjoyment	(*a*)	0.625	0.082	7.643	<0.001	0.583	0.482	0.685
Enjoyment → Engagement	(*b*)	0.458	0.086	5.311	<0.001	0.663	0.509	0.817
Positive → Engagement	(*c*′)	0.176	0.054	3.292	<0.001	0.239	0.099	0.378
Positive → Enjoyment → Engagement	(*a* * *b*)	0.286	0.056	5.148	<0.001	0.387	0.271	0.502
Positive → Engagement	(*c*)	0.462	0.064	7.265	<0.001	0.626	0.517	0.733

Positive, Positive Coach Behavior, Enjoyment, Sport Enjoyment, Engagement, Sport Engagement.

A positive and moderate direct effect of positive coaching behaviors on sport engagement was found (*βc*′ = 0.239 [0.10, 0.38]; *z* = 3.29, *p* < 0.001). A one-unit increase in positive coaching behaviors was directly associated with an increase of *B*(*c*′) = 0.176 points (SE = 0.054) in sport engagement. A positive and moderate indirect effect of positive coaching behaviors on sport engagement was also found (*β*_(*ab*)_ = 0.387 [0.27, 0.50]; *z* = 5.15, *p* < 0.001). A one-unit increase in positive coaching behaviors was indirectly associated with an increase of *B*_(*ab*)_ = .286 points (SE = 0.056) in sport engagement through enjoyment in sport.

The total effect of positive coaching behaviors on sport engagement, both directly and indirectly (through enjoyment in sport), was found to be positive and highly significant (*β_c_* = 0.626 [0.52, 0.73]; *z* = 7.26, *p* < 0.001). A one-unit increase in positive coaching behaviors was associated with a total increase of *B*_(*c*)_ = 0.462 points (SE = 0.064) in sport engagement. The findings indicate that enjoyment in sport plays a partial mediating role in the relationship between positive coaching behaviors and sport engagement. Part of the effect of positive coaching behaviors on sport engagement occurs through enjoyment in sport.

**Figure 2:**
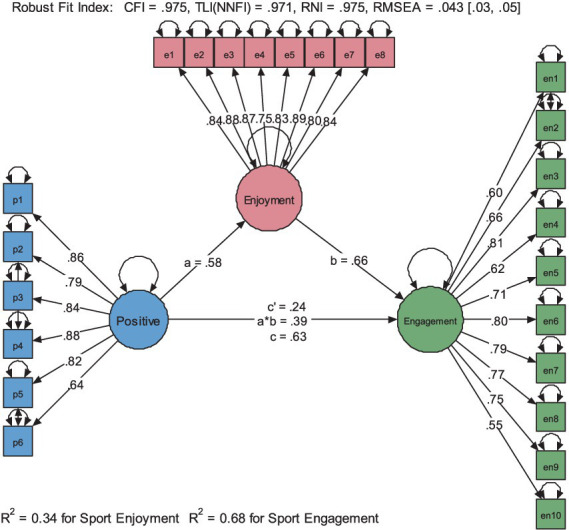
Path diagram for structural regression modelling.

The confidence intervals of all paths in the model do not include zero, confirming that the effects are statistically significant. The model explains approximately 34% of the variance in enjoyment (*R*^2^ = 0.340, Adj. *R*^2^ = 0.339) and approximately 68% of the variance in sport engagement (*R*^2^ = 0.681, Adj. *R*^2^ = 0.680). The standardized path coefficients of the tested structural regression model are presented in Figure 2. The acceptance–rejection status of the hypotheses based on the structural model results is summarized in Table 5. As shown in Table 5, all hypotheses were accepted, and sport enjoyment played a partial mediating role in the relationship between positive coaching behaviors and sport engagement.

**Table 5:** Acceptance-rejection status of hypotheses.

Hypothesis	Dependent	Independent	Mediator	Effect type	*p* value	Decision
H1	S. Engagement	PCB	–	Direct effect (*c*′)	<0.001	Accept
H2	S. Enjoyment	PCB	–	Direct effect (*a*)	<0.001	Accept
H3	S. Engagement	Sport enjoyment	–	Direct effect (*b*)	<0.001	Accept
H4	S. Engagement	PCB	S. Enjoyment	Indirect effect (*a* * *b*)	<0.001	Accept/PM

S., Sport, PCB, Positive Coach Behaviours, PM, Partial mediation, WM, Without mediation.

According to Table 5, all four hypotheses were accepted. The significant direct and indirect effects indicate that positive coaching behaviors were associated with sport engagement both directly and indirectly through sport enjoyment, supporting the partial mediation model.

## 4 Discussion

The present study examined the relationship between positive coaching behaviors and sport engagement, as well as the mediating role of enjoyment in sport in this relationship. The findings demonstrated that the hypotheses formulated within the scope of the study were supported. Accordingly, positive coaching behaviors were found to be positively associated with both enjoyment in sport and sport engagement. Furthermore, enjoyment in sport was found to be positively related to sport engagement and to play a mediating role in the relationship between positive coaching behaviors and sport engagement.

### 4.1 Relationship between positive coaching behaviors and sport engagement (H1)

The analyses revealed a positive and significant relationship between positive coaching behaviors and sport engagement (*β* = 0.239, *p* < 0.001). This finding suggests that supportive, constructive, and autonomy-supportive behaviors perceived by athletes from their coaches are positively associated with their levels of sport engagement. Evaluated within the framework of Self-Determination Theory, it may be argued that the autonomy, competence, and relatedness support provided by coaches may reinforce athletes’ motivational processes, which in turn may be associated with psychological engagement to sport (Deci and Ryan, 2000). In this respect, the present finding is consistent with studies demonstrating that supportive and autonomy-supportive coaching behaviors are associated with athletes’ motivational processes and sport commitment. Indeed, Altın et al. (2023) reported that positive coaching behaviors were associated with athletes’ commitment levels; Pulido et al. (2018) found that supportive coaching behaviors were positively linked to intrinsic motivation and sport commitment processes; and O’Neil and Hodge (2020) and Hodge et al. (2023) reported that autonomy-supportive coaching approaches may be associated with athletes’ commitment indicators. Similarly, Marholz et al. (2016) and Murillo et al. (2022) demonstrated that the motivational climate created by coaches was closely related to athletes’ sport commitment. In addition, there are systematic reviews and recent studies indicating that supportive leadership style and a positive motivational climate may be associated with athletes’ sport commitment processes (Merdan and Çağlar, 2022; Bae, 2023; Mohebi et al., 2024). Taken together, these findings support the relationship between positive coaching behaviors and sport engagement in a sample of elite young wrestlers.

### 4.2 Relationship between positive coaching behaviors and enjoyment in sport (H2)

The analyses revealed a positive and significant relationship between positive coaching behaviors and enjoyment in sport (*β* = 0.583, 95% CI [0.482, 0.685], *p* < 0.001). From a theoretical perspective, it is proposed that supportive social environments may be associated with athletes’ motivational and emotional experiences, which in turn may be linked to positive expehriences such as enjoyment in sport (Deci and Ryan, 2000).

In this respect, the present finding is consistent with studies demonstrating that the quality of the athlete–coach relationship is closely related to athletes’ motivational and emotional experiences. Adie and Jowett (2010) reported that relationship quality was linked to athletes’ motivational processes; Kim et al. (2021) found that supportive coaching behaviors were associated with enjoyment in sport and the intention to continue in sport. Similarly, Álvarez et al. (2009) and Quested et al. (2013) demonstrated that coach support may be linked to athletes’ positive sport experiences. Moreover, Mageau and Vallerand (2003) noted that autonomy-supportive coaching behaviors may foster athletes’ intrinsic motivation; and Young and Medic (2011) found that perceived social support may be associated with athletes’ levels of enjoyment in sport. In this context, the findings support that positive coaching behaviors constitute one of the important variables associated with enjoyment in sport among elite young wrestlers.

### 4.3 Relationship between enjoyment in sport and sport engagement (H3)

The analyses revealed a positive and significant relationship between enjoyment in sport and sport engagement (*β* = 0.663, 95% CI [0.509, 0.817], *p* < 0.001). This finding demonstrates that the enjoyment athletes derive from the sport experience is closely related to their level of psychological commitment to sport. Finding the sport experience enjoyable may increase individuals’ desire to continue this experience, thereby contributing to the development of a stronger psychological commitment to sport. Indeed, the literature emphasizes that enjoyment in sport is one of the strongest determinants of sport commitment.

Scanlan et al. (2003) demonstrated that enjoyment in sport plays a central role in commitment processes within the Sport Commitment Model. Similarly, Weiss et al. (2001) indicated that enjoyment in sport is one of the strongest predictors of sport commitment. Furthermore, studies by Garcia-Mas et al. (2010) and Choi and Kwon (2016) show that athletes with higher levels of enjoyment in sport tend to maintain their sport participation and exhibit higher levels of commitment. Asghari et al. (2022) reported that enjoyment in sport is one of the key variables associated with sport motivation and commitment. Comparable findings have also been reported in studies of youth basketball players. Soares et al. (2020b) found that young female basketball players reported high levels of sport enjoyment across different competitive levels, although the sources of enjoyment differed according to competitive level. The authors also suggested that structured training and competition environments may support athletes’ sport participation and commitment. Similarly, Soares et al. (2025) showed that sources of enjoyment may change over time and differ across sports, emphasizing the developmental nature of enjoyment and its sensitivity to sport-specific conditions. In addition, Soares et al. (2020a) found that persistence and dropout in youth basketball were associated with motivational, developmental, and performance-related characteristics, indicating that continued sport participation is shaped by multiple interacting factors. These findings demonstrate that enjoyment in sport is not merely an emotional outcome but also a fundamental determinant that shapes sport commitment, and they support the conclusion that the present research findings are generally consistent with the literature.

### 4.4 Mediating role of enjoyment in sport (H4)

The analyses demonstrated that enjoyment in sport plays a significant mediating role in the relationship between positive coaching behaviors and sport engagement (ab: *β* = 0.387, 95% CI [0.271, 0.502], *p* < 0.001). The obtained results indicate that coaching behaviors in the sport environment generate not only direct behavioral outcomes but also indirect effects through emotional experiences. In particular, it may be argued that supportive and development-oriented coaching behaviors contribute to athletes’ more positive perception of the sport experience, thereby increasing their level of enjoyment in sport and strengthening their commitment to sport. Similar findings have been reported in the literature. Weiss et al. (2001), in a study conducted within the Sport Commitment Model framework, demonstrated that enjoyment in sport is one of the strongest determinants of sport commitment and may assume an explanatory role in the commitment process. Studies by Scanlan et al. on the Sport Commitment Model reveal that enjoyment in sport is addressed as a central component in commitment processes.

Similarly, Bandura and Kavussanu (2018) noted that athletes’ perception of their coach as an authentic leader was positively associated with both enjoyment in sport and sport commitment, and that this relationship could be explained through psychosocial processes such as trust and autonomy. Furthermore, Braga-Pereira et al. (2024) demonstrated that supportive coaching behaviors, enjoyment in sport, and motivation may be associated with sport commitment indicators over time. Reynders et al. (2019) showed that a need-supportive coaching style was associated with higher autonomous motivation and commitment in athletes, thus providing indirect evidence that supportive coaching approaches may be linked to sport commitment through positive psychological processes. The obtained results demonstrate that positive coaching behaviors are associated with sport engagement both directly and indirectly through enjoyment in sport.

The exclusive inclusion of young elite wrestlers should be considered when interpreting the findings. Because the sample comprised athletes who were actively competing and had remained within the elite sport system, wrestlers with lower sport enjoyment, weaker engagement, or negative sport experiences may have discontinued participation and were therefore not represented. Accordingly, the observed associations may partly reflect the characteristics of athletes who continued their involvement in competitive wrestling.

### 4.5 Conclusion

The present study demonstrates that sport engagement in young elite wrestlers is not merely a behavioral process but is also closely related to the emotional quality of the sport experience. The findings reveal that positive coaching behaviors are associated with sport engagement both directly and indirectly through enjoyment in sport. This result suggests that supportive coaching behaviors may play an important role in athletes’ perception of sport as a more positive and worthwhile experience. For this reason, enjoyment in sport must be taken into consideration alongside coaching behaviors in understanding sport engagement.

From a practical perspective, the findings suggest the importance of creating training environments that support not only performance outcomes but also athletes’ sport enjoyment and engagement. Providing constructive feedback, recognizing effort and individual development, and involving athletes in appropriate decision-making processes may contribute to this aim. Sport managers may also incorporate positive and autonomy-supportive coaching practices into coach education programs and consider athletes’ enjoyment, engagement, and continued participation alongside sporting success when evaluating coaching environments. Given the cross-sectional design of the study, these implications should be interpreted as evidence-informed practical considerations rather than causal recommendations.

### 4.6 Limitations

This study should be evaluated within certain limitations. First, because the study is based on a cross-sectional design, the relationships among variables should not be interpreted causally. In addition, data were collected through self-report measures; this may introduce limitations arising from response patterns based on participants’ perceptions. Because the study group consisted exclusively of elite-level male wrestlers aged 18–21, caution should be exercised when generalizing the findings to different age groups, female athletes, or different sport disciplines. Moreover, because the sample consisted only of wrestlers who were actively competing and had remained within the elite sport system, individuals who had discontinued wrestling or no longer competed at the elite level were not represented. This may have introduced a selection-related limitation and restricted the extent to which the findings can be generalized to wrestlers with different participation trajectories. Furthermore, the determination of the sample through purposive sampling constitutes another factor limiting the generalizability of the findings.

### 4.7 Theoretical and practical implications

This study contributes theoretically by examining the relationships among positive coaching behaviors, sport enjoyment, and sport commitment within the same model. The findings suggest that Self-Determination Theory, which proposes that supportive coaching behaviors are associated with athletes’ motivational and emotional experiences, and the Sport Commitment Model, which considers sport enjoyment one of the fundamental sources of sport commitment, may be usefully evaluated together. The significant mediating effect of sport enjoyment in the relationship between positive coaching behaviors and sport commitment provides an explanatory framework for understanding the psychological process through which the social and motivational environment created by the coach may be associated with sport commitment. Nevertheless, owing to the cross-sectional design of the study, these findings should be interpreted as associations among the variables rather than as causal effects.

From a practical perspective, the findings indicate that effective coaching is not limited to guiding athletes’ technical, tactical, and physical development. Coaches working with young elite wrestlers may contribute to creating a more supportive and enjoyable sport environment by listening to athletes, valuing their efforts and individual development, treating them fairly and respectfully, and providing constructive feedback in response to mistakes. Therefore, coach education programs may benefit from placing greater emphasis on autonomy-supportive communication, constructive feedback, athlete-centered approaches, and the development of positive coach–athlete relationships. Sport psychologists, clubs, and federations may also consider sport enjoyment not merely as an indicator of satisfaction but as an important psychological indicator associated with sport commitment processes.

### 4.8 Future research recommendations

The present study’s findings demonstrate that positive coaching behaviors are associated with athletes’ enjoyment in sport and sport engagement processes. In light of these findings, the following recommendations may be offered for future research. The present model addressed only positive coaching behaviors. Future studies may incorporate additional variables such as athletes’ personality characteristics, emotional intelligence, motivation types, perceived social support levels, and team identity into the model to examine the relationships more comprehensively. The present study was conducted based on a quantitative method. However, qualitative research, mixed-method designs, and different modeling approaches may also be employed to achieve a more in-depth understanding of the relationships among coaching behaviors, enjoyment in sport, and sport engagement. The present study is limited to a specific age group and sport discipline. Conducting similar studies with different age groups, different performance levels, and different sport disciplines may increase the generalizability of the findings. Future studies should include wrestlers who have discontinued participation in competitive wrestling and examine the reasons underlying their withdrawal from the sport. Comparisons between these individuals and wrestlers who remain actively involved in competitive wrestling may provide a clearer understanding of the factors associated with sustained sport engagement and withdrawal from the sport. The present study focused on positive coaching behaviors. In future research, the examination of different dimensions of coaching behaviors (e.g., supportive and controlling approaches) together in a comparative manner may contribute to a more holistic understanding of the athlete experience. These recommendations will contribute to better understanding athletes’ sport commitment processes and evaluating the long-term effects of coach–athlete relationships in future research.

## Data Availability

The raw data supporting the conclusions of this article will be made available by the authors, without undue reservation.
